# Silhouette Analysis for Performance Evaluation in Machine Learning with Applications to Clustering

**DOI:** 10.3390/e23060759

**Published:** 2021-06-16

**Authors:** Meshal Shutaywi, Nezamoddin N. Kachouie

**Affiliations:** 1 Department of Mathematics, King Abdulaziz University, Rabigh 21911, Saudi Arabia; mshutaywi@kau.edu.sa; 2 Department of Mathematical Sciences, Florida Institute of Technology, Melbourne, FL 32901, USA

**Keywords:** k-means, kernel k-means, machine learning, nonlinear clustering, silhouette index, weighted clustering

## Abstract

Grouping the objects based on their similarities is an important common task in machine learning applications. Many clustering methods have been developed, among them k-means based clustering methods have been broadly used and several extensions have been developed to improve the original k-means clustering method such as k-means ++ and kernel k-means. K-means is a linear clustering method; that is, it divides the objects into linearly separable groups, while kernel k-means is a non-linear technique. Kernel k-means projects the elements to a higher dimensional feature space using a kernel function, and then groups them. Different kernel functions may not perform similarly in clustering of a data set and, in turn, choosing the right kernel for an application could be challenging. In our previous work, we introduced a weighted majority voting method for clustering based on normalized mutual information (NMI). NMI is a supervised method where the true labels for a training set are required to calculate NMI. In this study, we extend our previous work of aggregating the clustering results to develop an unsupervised weighting function where a training set is not available. The proposed weighting function here is based on Silhouette index, as an unsupervised criterion. As a result, a training set is not required to calculate Silhouette index. This makes our new method more sensible in terms of clustering concept.

## 1. Introduction

There is a high demand for developing new methods to discover hidden structures, identify patterns, and recognize different groups in machine learning applications [1]. Cluster analysis has been widely applied for dividing objects into different groups based on their similarities [2]. Cluster analysis is an important task in different areas of study such as medical diagnosis, information retrieval, marketing, and social sciences [3]. For instance, in medical diagnosis, clustering can help to divide the patients into different groups based on the stage of the disease and in marketing, clustering can assist in determining customers with similar interests to customize the advertisement process and make it more effective [4].

Cluster analysis is an unsupervised learning method [5] to optimize an objective function based on features similarities [6]. The goal is finding groups, such that elements in the same group have similar features. Clustering algorithms often use a search method to optimize the objective function. To quantify the distinctiveness of each pair of elements, a distance measure such as Euclidean distance or Manhattan distance can be used. The number of clusters is often unknown and must be chosen for cluster analysis. Hence, either the number of clusters is specified by the user or it must be estimated using an overall distance measure such as the sum of the squared distances of elements from their cluster centers. An objective function is optimized by minimizing the distance of elements to their cluster centers (within-cluster distance) and/or maximizing the distance between cluster centers (between-cluster distance). A search strategy is required to find the groups that optimize the objective function [1].

Several clustering methods have been developed [7], such as k-means, model-based clustering, spectral clustering, and Hierarchical clustering. The focus of this study is on the k-means based clustering methods. K-means is a well-known clustering method with the aim of minimizing the Euclidean distance between each point and the center of the cluster to which it belongs. Advantages of k-means are its simplicity and speed [8]. However, k-means can only discover clusters that are linearly separable. In contrast, kernel k-means is a non-linear extension of the k-means clustering method that can identify clusters that are not linearly separable. Although objective functions of both methods are similar, elements are projected into a higher dimensional space in kernel k-means. 

In our previous work [9], we introduced a weighted majority voting method for clustering based on normalized mutual information (NMI). We showed that the clustering results highly depend on the selected kernel function when using kernel k-means method. For example, by choosing a different kernel such as Gaussian, polynomial, or hyperbolic tangent kernels, we obtain different clustering results for the same dataset. Therefore, to eliminate the partiality based on the chosen kernel for an arbitrary application, we aggregated the clustering results obtained by different kernels. To achieve this, we implemented a weighting function to assign the weights to each kernel based on their performances. The performance of each kernel for clustering application was assessed by normalized mutual information (NMI) [10]. NMI is a supervised method, i.e., we need a training set for calculating NMI to evaluate the performance of a clustering method. However, a training set might not be available for the clustering purposes. Therefore, in this study, we extend our previous work of ensemble clustering by developing a weighting function that does not need a training set. Silhouette index is an unsupervised method for evaluating the performance of a clustering method [11]. Since the Silhouette index does not need a training set to evaluate the clustering performance, it is more relevant to the clustering concept. 

Here in this work, we have developed a different weighting function based on the Silhouette index. The paper is organized as follows. We review k-means, kernel k-means, and the Silhouette index in Section 2. Section 3 explains the proposed weighting function using the Silhouette index. Simulation studies and results are presented in Section 4, and the conclusions are provided in Section 5.

## 2. Background 

A brief review of k-means, kernel k-means, and the Silhouette index follow. 

### 2.1. K-Means

K-means choose *K* centers such that the total squared distances of each point and its cluster center is minimized. K-means technique can be summarized by first selecting *K* arbitrary centers, which are usually, as Lloyd’s algorithm suggests, uniformly selected at random from the data. Second, we must calculate the Euclidean distance between each element and all cluster centers separately, and assign each element to its closest cluster center. Third, new cluster centers are obtained by averaging the Euclidean distances of all elements grouped in the same cluster in Step 2. Finally, the second and the third steps are repeated until the algorithm converges, i.e., cluster centers obtained in current iteration are the same or very close to the cluster centers obtained in the previous iteration. K-means objective functions can be written as $\sum_{k=1}^{K}\sum_{x_{i}\in \;\pi_{k}}\|x_{i}-\mu_{k}\|{}^{2}$, where $\pi_{k}$ is cluster k, $\mu_{k}$ is the center of cluster k, and ‖⋅‖ is the Euclidean distance. 

### 2.2. Kernel K-Means

Kernel k-means is an extension of k-means for grouping objects that are not linearly separable. The idea of kernel k-means clustering relies on projecting the elements into a higher-dimensional feature space using a non-linear function to make them linearly separable in the projected space. The kernel k-means algorithm is summarized below [10].

Let $\left\{x_{1},x_{2},\ldots ,x_{n}\right\}$ be a set of *n* data points, *K* be the number of clusters, $\pi_{k}$ be the cluster k, ${\left\{\pi_{k}\right\}}_{k=1}^{K}$ be a partitioning of points into *K* groups, and ϕ be a non-linear function projecting each data point $x_{i}$ to a higher dimensional space. Each element in the kernel matrix M is:

(1) $$M_{ij}=\phi \left(x_{i}\right)\cdot \phi \left(x_{j}\right),\;\mathrm{for}\;i,j=1,2,\ldots ,\;n.$$

where $\phi \left(x_{i}\right)$ and $\phi \left(x_{j}\right)$ are transformations of $x_{i}$ and $x_{j}$ respectively. Some popular kernel functions are the Radial Basis Function known as the Gaussian kernel [12], polynomial kernel, and sigmoid kernel. The procedure is similar to k-means, but in the transformed space as follows. Step 1: after transforming the data points into the new space, each cluster center $\mu_{k}$ must be randomly initialized. Step 2: the Euclidean distance between each element and all cluster centers $\mu_{k}$’s is computed in the transformed space by:

(2) $$\phi \left(x_{i}\right)-\mu_{k}=\phi \left(x_{i}\right)-\underset{x_{i}\in \pi_{k}}{\sum }\frac{\phi \left(x_{i}\right)}{\left|\pi_{k}\right|}=\phi \left(x_{i}\right)\cdot \phi \left(x_{i}\right)\;-\frac{2\sum_{x_{j}\in \pi_{k}}\phi \left(x_{i}\right)\cdot \phi \left(x_{j}\right)}{\left|\pi_{k}\right|}+\frac{2\sum_{x_{j},x_{c}\in \pi_{k}}\phi \left(x_{j}\right)\cdot \phi \left(x_{c}\right)}{{\left|\pi_{k}\right|}^{2}}.$$

where $\left|\pi_{k}\right|$ is the number of elements in cluster $\pi_{k}$. Step 3: each data point in the transformed space is assigned to the closest cluster with minimum distance in the transformed space. Step 4: a new cluster center $\mu_{k}$ is obtained for cluster k by averaging Euclidean distance of all elements that were assigned to cluster $\pi_{k}$ in the transformed space in the previous iteration:

(3) $$\mu_{k}=\underset{x_{i}\in \pi_{k}}{\sum }\frac{\phi \left(x_{i}\right)}{\left|\pi_{k}\right|},\;\;\mathrm{for}\;k=1,2,\ldots ,K.$$

Steps 2 to 4 will be repeated to minimize the objective function:

(4) $$D\left({\left\{\pi_{k}\right\}}_{k=1}^{K}\right)=\overset{K}{\underset{k=1}{\sum }}\underset{x_{i}\in \pi_{k}}{\sum }\|\phi \left(x_{i}\right)-\mu_{k}\|{}^{2}.$$

The algorithm will converge when the obtained cluster centers in the current iteration are the same as or very close to the cluster centers obtained in the previous iteration.

### 2.3. Silhouette Index

**Figure 1:**
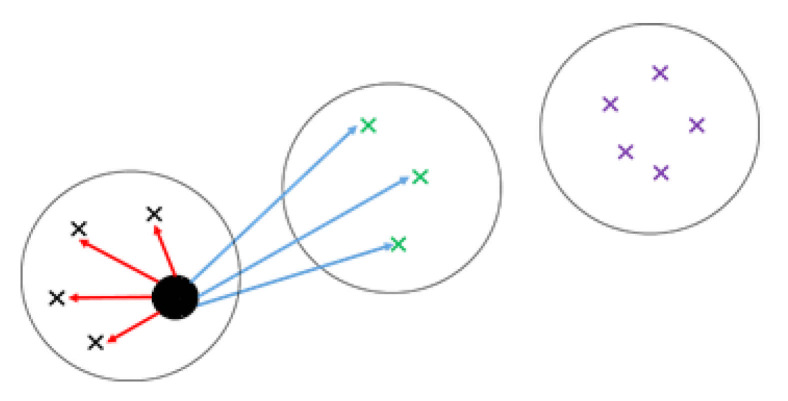
Procedure to calculate Silhouette index for a typical data point X. Red lines show the distances between X and every data point clustered in the same group. Blue lines show the distances between X and every data point clustered in the nearest group.

There are several methods to evaluate clustering results, such as the Rand index [13], adjusted Rand index [14], distortion score [11], and Silhouette index. While most of the performance evaluation methods need a training set, the Silhouette index does not need a training set to evaluate the clustering results. This makes it more appropriate for a clustering task. In this work, we use the Silhouette index to evaluate the clustering performance. The Silhouette width $s\left(x_{i}\right)$ for the point $x_{i}$ is defined as [11]:

(5) $$s\left(x_{i}\right)=\frac{b\left(x_{i}\right)-a\left(x_{i}\right)}{\max\left\{b\left(x_{i}\right),\;a\left(x_{i}\right)\right\}}.$$

where $x_{i}$ is an element in cluster $\pi_{k}$, $a\left(x_{i}\right)$ is the average distance of $x_{i}$ to all other elements in the cluster $\pi_{k}$ (within dissimilarity), and

$$b\left(x_{i}\right)=\min\;\left\{d_{l}\left(x_{i}\right)\right\},\;\mathrm{among}\;\mathrm{all}\;\mathrm{clusters}\;l\neq k.$$

where $d_{l}\left(x_{i}\right)$ is the average distance from $x_{i}$ to all points in cluster $\pi_{l}$ for l≠k (between dissimilarity). From Equation (5) the value of the Silhouette width can vary between −1 and 1. A negative value is undesirable because it is related to a case in which $a\left(x_{i}\right)$ is greater than $b\left(x_{i}\right)$, and the means within dissimilarity is greater than between dissimilarity. A positive value is obtained where $a\left(x_{i}\right)<b\left(x_{i}\right)$, and the Silhouette width reaches its maximum $s\left(x_{i}\right)=1$ for $a\left(x_{i}\right)=0$ [11]. The greater the (positive) $s\left(x_{i}\right)$ value of an element, the higher the likelihood to be clustered in the correct group. Elements with negative $s\left(x_{i}\right)$ are more likely to be clustered in wrong groups [15]. A typical example is shown in Figure 1, where $x_{i}$ is shown by a black disk, $a\left(x_{i}\right)$ by red lines, and $b\left(x_{i}\right)$ by blue lines. The average Silhouette width for a cluster is the average $s\left(x_{i}\right)$ for all points in the cluster, and the average Silhouette width for the entire clustering result is the average $s\left(x_{i}\right)$ of all points in every cluster. We discuss the proposed weighting function using Silhouette in detail in the next section.

## 3. Weighted Clustering Method Using Silhouette Index

The goal of the proposed method is to assess the performance of different clustering methods and combine their results based on their performances to provide a single outcome. The focus here is on kernel k-means, because selecting the right kernel function $F\left(x_{i},x_{j}\right)=\phi \left(x_{i}\right)\cdot \phi \left(x_{j}\right)$ for an arbitrary application is not obvious. Therefore, applying a set of different kernels for clustering and aggregating the clustering results based on the performance of different kernels can provide consistent results and eliminate the partiality of the results based on the selected kernel. We compute the performance of a kernel by computing the average Silhouette width (γ) for the clustering results obtained by the kernel. Three kernels including Gaussian, polynomial, and hyperbolic tangent are used to project the elements as follows. 

Gaussian kernel with standard deviation σ is given by:

(6) $$\phi \left(x_{i}\right)\cdot \phi \left(x_{j}\right)=e^{\frac{-\|x_{i}-x_{j}\|{}^{2}}{2\sigma^{2}}}.$$

Polynomial kernel is defined by:

(7) $$\phi \left(x_{i}\right)\cdot \phi \left(x_{j}\right)={\left(px_{i}{}^{T}x_{j}+c\right)}^{l}.$$

where p is the slope, c is the intercept, and l is the polynomial order.

Hyperbolic tangent kernel is given by:

(8) $$\phi \left(x_{i}\right)\cdot \phi \left(x_{j}\right)=\tanh\;\left(px_{i}^{T}\;x_{j}+c\right).$$

where p is slope and c is the intercept. 

We compute the average Silhouette width for the clustering results obtained by each kernel separately. We then combine the results using computed weights for each kernel. Let $\gamma_{j}$ be the average Silhouette width for the clustering result obtained using the *j*th kernel. The assigned weight $\delta_{j}$ for the clustering result of the *j*th kernel is computed by:

(9) $$\delta_{j}=\frac{\gamma_{j}+1}{\sum_{m=1}^{d}(\gamma_{m}+1)},\;\;\;\mathrm{for}\;j=1,\;2,\cdots ,d.$$

where *d* is the number of kernel functions (here *d* = 3) and

(10) $$\overset{d}{\underset{j=1}{\sum }}\delta_{j}=1.$$

The Silhouette value is used to evaluate and assign weight to each kernel. Weights must be non-negative real values between 0 and 1 and must sum up to one. Hence, we shifted the Silhouette scores by adding one, obtaining shifted Silhouette scores ranging from 0 to 2. Normalized non-negative weights are consequently computed by dividing the shifted Silhouette scores to the sum of shifted Silhouette scores. Next, for each data point, we sum up the weights ($\delta_{j}$) assigned to the kernels that clustered the data point into the same group. To ensure the consistency of the group labels by different kernels, the clusters found by the first kernel are considered the base group labels. The Euclidean distance between the cluster centers found by each kernel and the cluster centers found by the first kernel (reference groups) are computed to assign the consistent cluster labels. The cluster labels are then preserved based on the minimum sum of Euclidean distances. Finally, for a given data point, we compare the total weights computed for each cluster label, and the data point will be considered in the group with the highest weight. The proposed method is summarized below.

1. Let $\Omega_{d}$ be a set of d kernel functions $h_{1}$ to $h_{d}$. Perform kernel k-means method using kernels in the kernel set and generate d clustering results $\begin{array}{cc} \Gamma_{j}\;,\;\mathrm{for}\; & j=\left\{1,\;2,\;\ldots ,d\right\} \end{array}$;
2. Compute average Silhouette width $\gamma_{j}$ for clustering results $\begin{array}{cc} \Gamma_{j}\;,\;\mathrm{for} & j=\left\{1,\;2,\;\ldots ,\;d\right\} \end{array}$ obtained by kernel $h_{j}$ for all kernels $j=\left\{1,\;2,\;\ldots ,\;d\right\}$;
3. Shift Silhouette values from {−1 to 1} to {0 to 2} to compute non-negative weights $\delta_{j}$ for each kernel;
4. For each data point, use the computed weights $\delta_{j}$ in step (3) to combine the clustering results $\begin{array}{cc} \Gamma_{j}\;,\;\mathrm{for} & j=\left\{1,\;2,\;\ldots ,\;d\right\} \end{array}$ as follows:
  - Sum up the weights corresponding to the kernels that assign the same cluster label to the data point;
  - Compare the total weight of each cluster for the data point;
  - Group the data point to the cluster with the highest total weight.

## 4. Simulation

To evaluate the performance of the proposed method, we have applied it to several benchmark datasets as follow.

- Bensaid data: it contains 49 two-dimensional elements grouped in three classes [16].
- Dunn data: it contains 90 two-dimensional elements grouped in two classes [17].
- Iris data: it contains 150 four-dimensional elements grouped in three classes [18].
- Seed data: it contains 210 seven-dimensional elements grouped in three classes [19].

To address the random initialization of the cluster centers in kernel k-means, we applied the proposed method in a Monte Carlo setting and averaged the results obtained through 100 Monte Carlo trials. In each trial, initial cluster centers were randomly generated. For each dataset, kernel k-means with three different kernel functions (Gaussian, polynomial, and hyperbolic tangent) were used. To evaluate the clustering performance of each kernel, identified clusters by these three kernels are matched using their cluster centroids. Clustering performance of each kernel is then evaluated using the Monte Carlo average of the Silhouette index. Next, weight of each cluster is assigned by the estimated performance of the kernel. Finally, clustering results of three kernels are merged using majority voting and the proposed weighted majority voting method. 

## 5. Results

In this section, the clustering results obtained using the proposed method are demonstrated. Table 1 summarizes the Monte Carlo average for the average Silhouette indices, and the Monte Carlo average of true rates for the clustering results of each dataset obtained using Gaussian, polynomial, and tangent kernels along with combined results using the proposed weighted method. We use package “kernlab” in R, and hyper-parameters are set by default “automatic” setting that use a heuristic to determine suitable values for the hyper-parameters of the kernel.

**Figure 2:**
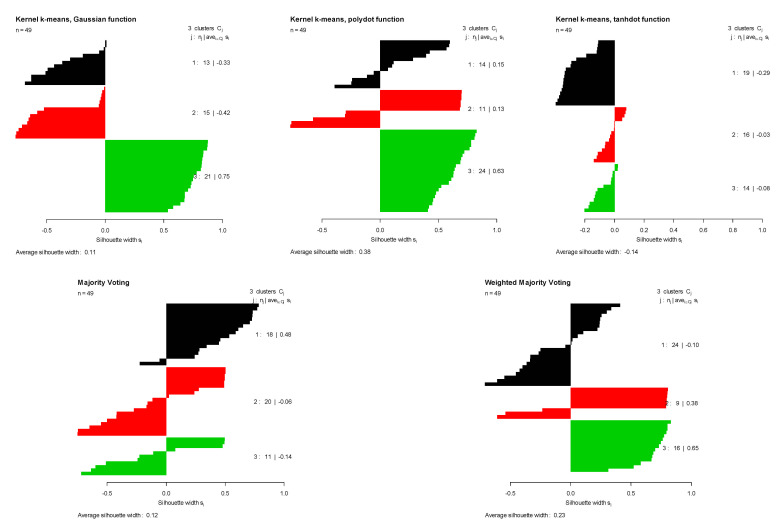
Silhouette index obtained for clustering results of Bensaid dataset using kernel k-means with three different kernel functions (Gaussian, polynomial, and tangent), majority voting, and the proposed weighted method.

**Figure 3:**
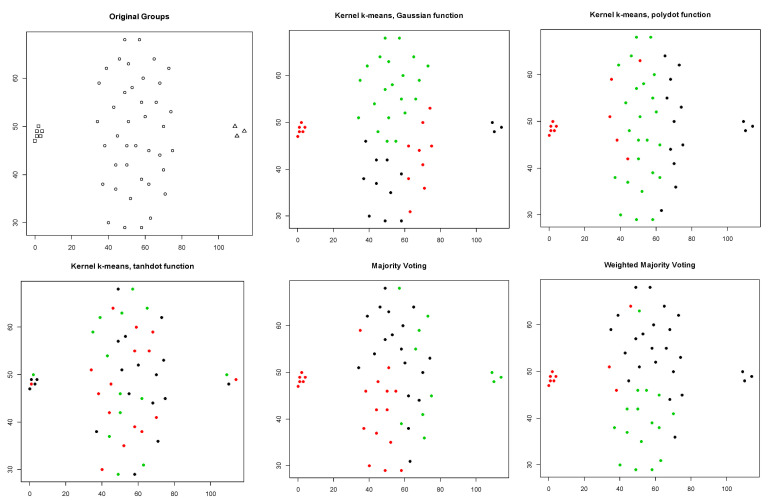
Original groups in the Bensaid data and the clustering results of kernel k-means, obtained using three different kernel functions (Gaussian, polynomial, and tangent), majority voting, and the proposed weighted method. Results are visualized using 1st and 2nd PCA.

The first row of Table 1 summarizes the clustering results for the Bensaid dataset. For this dataset, the polynomial kernel provided the best performance among all kernels based on the Monte Carlo average true rate (0.641) and Monte Carlo average of average Silhouette indices (0.453). Figure 2 (top left) demonstrates the three original groups in the Bensaid dataset along with the clustering results obtained by three different kernels, majority voting, and the proposed weighted method. It shows that among all kernels, the polynomial kernel could better distinguish the original clusters in the Bensaid dataset. The clustering results of these three kernels are combined using the calculated weights for each kernel. Then, the Monte Carlo average of average Silhouette indices and the Monte Carlo average of the true rates are obtained for both majority voting and the proposed method. The proposed method outperforms majority voting with the Monte Carlo average true rate of 0.611 in comparison with 0.592. Figure 3 shows the Silhouette index for all data points in the Bensaid dataset, computed for clustering results obtained using each kernel separately. A colored horizontal line shows the Silhouette index of each data element. We can see that there are smaller number of data points with negative Silhouette scores in the clustering results obtained by the proposed method in comparison with that of the majority voting method. As a result, the average Silhouette index of the proposed method is higher.

**Figure 4:**
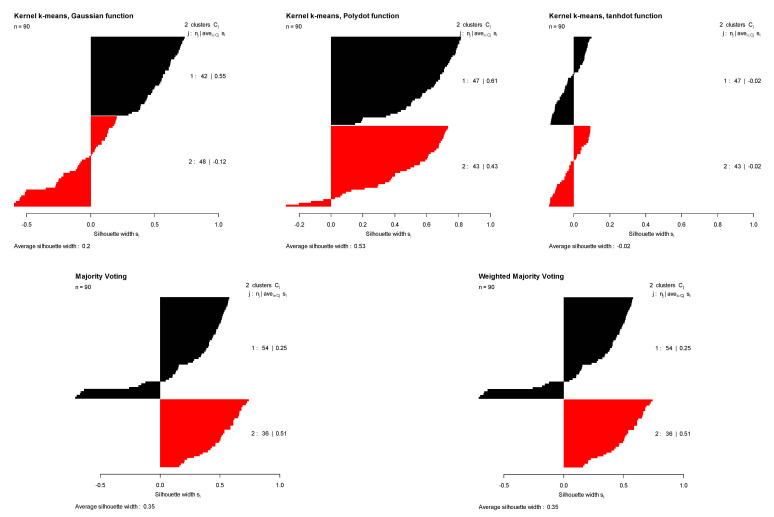
Silhouette index obtained for clustering results of the Dunn dataset using kernel k-means with three different kernel functions (Gaussian, polynomial, and tangent), majority voting, and the proposed weighted method.

**Figure 5:**
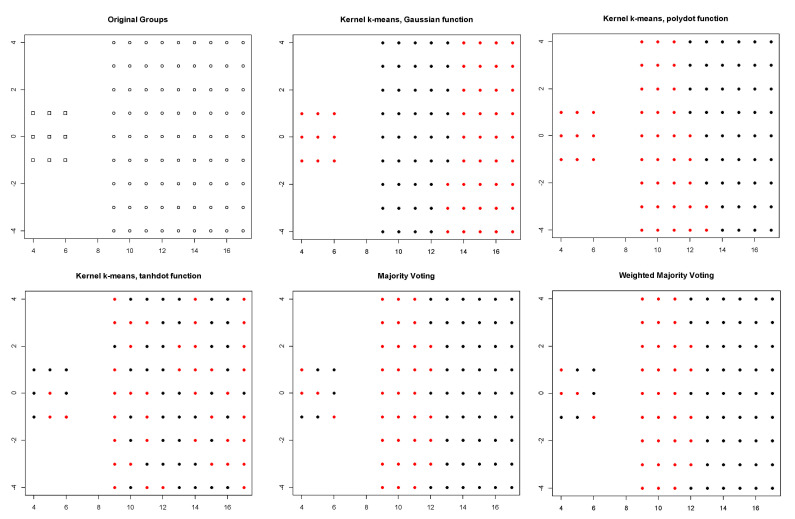
Original groups in the Dunn data and the clustering results of kernel k-means obtained using three different kernel functions (Gaussian, polynomial, and tangent), majority voting, and the proposed weighted method. Results are visualized using 1st and 2nd PCA.

The second row of Table 1 summarizes the clustering results for the Dunn dataset. For this dataset with two original groups, clustering performance of three different kernels were comparable with the Monte Carlo average true rates of 0.453, 0.521, and 0.417 for Gaussian, polynomial, and tangent kernels, respectively. Among them, polynomial kernels provided slightly better results (with the true rate of 0.521). As we can see in Table 1, the Monte Carlo average of the average Silhouette index for this kernel is also higher than those of the other two kernels (0.418), resulting a higher weight for this kernel when we combine the results. Figure 4 shows the original groups in the Dunn dataset and the clustering results obtained using kernel k-means with three different kernel functions, majority voting, and the proposed weighted method. Since three different kernels produced comparable results, the proposed method (weighted averaging) and majority voting (averaging) will also produce similar results, comparable with the results obtained by the original three kernels (Table 1). Figure 5 illustrates the Silhouette indices obtained for the clustering results of the Dunn dataset in a randomly selected trial (from 100 Monte Carlo trials).

**Figure 6:**
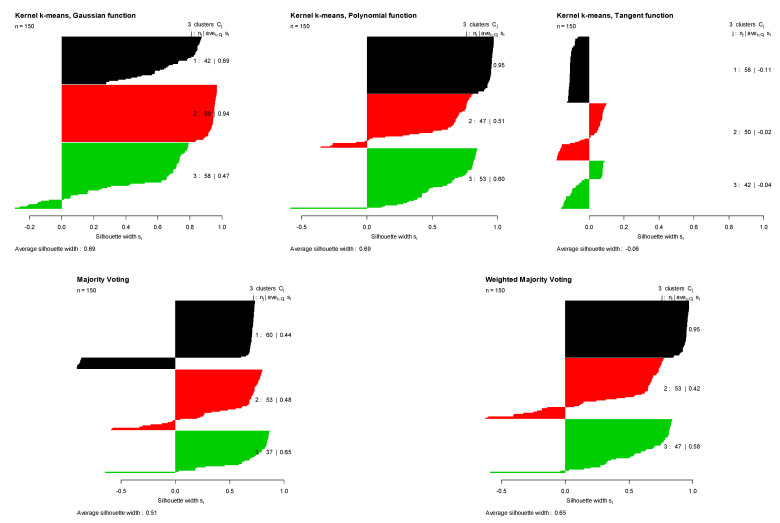
Silhouette index obtained for clustering results of the Iris dataset using kernel k-means with three different kernel functions (Gaussian, polynomial, and tangent), majority voting, and the proposed weighted method.

**Figure 7:**
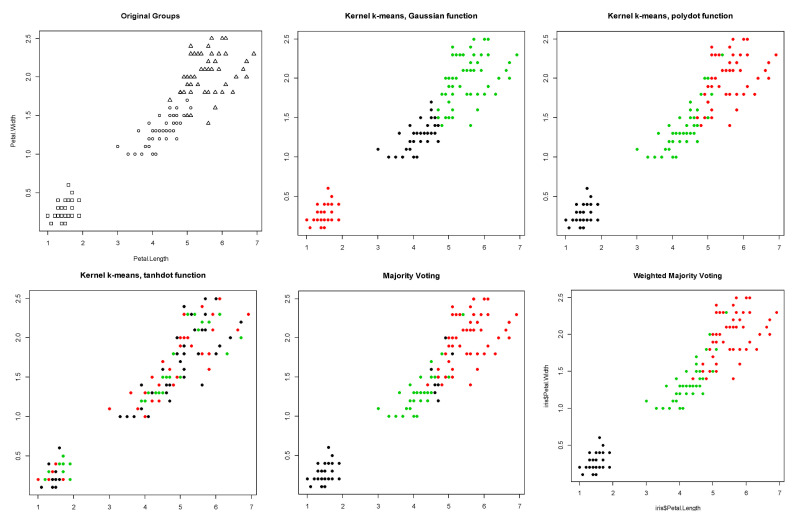
Original groups in the Iris data and the clustering results of kernel k-means obtained using three different kernel functions (Gaussian, polynomial, and tangent), majority voting, and the proposed weighted method. Results are visualized using 1st and 2nd PCA.

The third row of Table 1 summarizes the clustering results for the Iris dataset. For this dataset with three original groups, Gaussian kernels performed better than the other two kernels with a Monte Carlo average true rate of 0.856. Polynomial kernels produced comparable results to Gaussian kernels with a Monte Carlo average true rate of 0.850, while the Monte Carlo average true rate of the tangent kernel was 0.381. The computed Monte Carlo average Silhouette indices were 0.609, 0.609, and −0.068 for Gaussian, polynomial, and tangent kernels, respectively. As a result, Gaussian and polynomial kernels with the same high weight and tangent received a low weight in the proposed weighted method. The proposed method provided the Monte Carlo average true rate of 0.873, which is higher than Monte Carlo average true rates obtained by each kernel individually. As we can see in Table 1, the clustering results obtained by the proposed method also have higher Monte Carlo average true rates than the majority voting, since the latter assigns the same weights to each kernel. Figure 6 shows the original groups in the Iris dataset, and the clustering results obtained using kernel k-means with three different kernel functions, majority voting, and the proposed weighted method. Figure 7 shows the Silhouette indices calculated for the clustering results of Iris data obtained using kernel k-means with three different kernel functions, majority voting, and the proposed weighted method in a randomly selected trial (from 100 Monte Carlo trails). 

**Figure 8:**
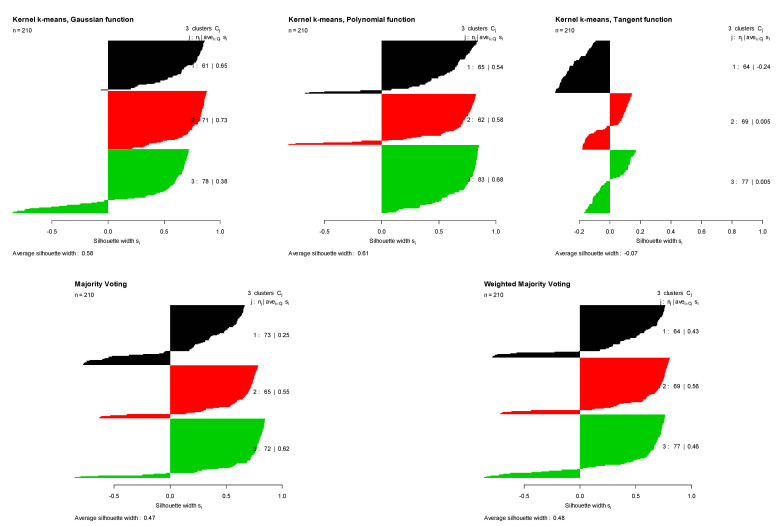
Silhouette index obtained for clustering results of the Seed dataset using kernel k-means with three different kernel functions (Gaussian, polynomial, and tangent), majority voting, and the proposed weighted method.

**Figure 9:**
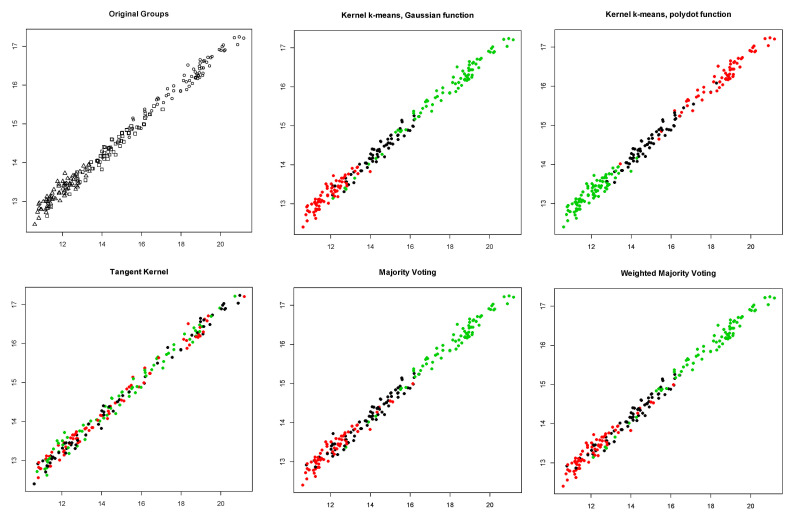
Original groups in the Seed data and the clustering results of kernel k-means obtained using three different kernel functions (Gaussian, polynomial, and tangent), majority voting, and the proposed weighted method. Results are visualized using 1st and 2nd PCA.

The fourth row of Table 1 summarizes the clustering results for the Seed dataset. For this dataset with three original groups, such as with the Iris data, the Gaussian kernel performed better than the other two kernels, with a Monte Carlo average true rate of 0.869. Polynomial kernels produced comparable results to Gaussian kernels with a Monte Carlo average true rate of 0.842, while the Monte Carlo average true rate of the tangent kernel was 0.376. As a result, Gaussian and polynomial kernels had high weights and the tangent received a low weight in the proposed weighted method. The proposed method obtained the Monte Carlo average true rate of 0.862, which is higher than that of the majority voting (0.852), and almost as high as the best performance obtained by the Gaussian kernel (0.869). Figure 8 shows the original groups in the Seed dataset, and the clustering results obtained using kernel k-means with three different kernel functions, majority voting, and the proposed weighted method. Figure 9 shows the Silhouette indices calculated for the clustering results of Seed data obtained using kernel k-means with three different kernel functions, majority voting, and the proposed weighted method in a randomly selected trial (from 100 Monte Carlo trails).

## 6. Conclusions and Future Research

In our previous work [9], we introduced a weighted mutual information method for aggregated kernel clustering. We showed that the clustering results highly depend on the selected kernel in the kernel k-means method. Therefore, we proposed a weighting function based on the clustering performance to aggregate the clustering results obtained by different kernels. The notion of aggregating the clustering results obtained by several kernel functions including Gaussian, polynomial, and hyperbolic tangent was based on assigning weight functions using their associated NMI values. Calculation of NMI requires a training set which might not be available, therefore in this paper, we extended our previous work of ensemble clustering by developing an unsupervised weighting function based on the Silhouette index. For calculating the Silhouette index, a training set is not required, which in turn is more sensible in the context of clustering. 

We applied the proposed method to different benchmark datasets and combined the results obtained by three different kernels. We should point out that the proposed aggregated method either improved the clustering performance or provided comparable results, depending on the performance of different kernels for the application at hand. However, the main goal of the proposed method is to obtain impartial results independent of the kernel function, rather than merely improving the clustering performance. The former is essential, because in the real applications the true results are not available to measure the clustering performance, and as a result the choice of the right clustering method is not obvious. In turn, by aggregating the results based on some sensible weights, the aggregated clustering results are less biased regarding the selected method. Here, we showed that not only the aggregated results are impartial where the ensemble performance was comparable with the best performance obtained by an individual kernel, but also, for some datasets, the aggregated results outperformed the best performance obtained by an individual kernel. The focus of future work is on further improvement of the performance of the ensemble clustering. As the average Silhouette score of the entire model demonstrates encouraging results, future research will be conducted to study a pointwise Silhouette score for potential further improvement. 

## Figures and Tables

**Table 1:** Monte Carlo average of the average Silhouette index (ASI) and Monte Carlo average of true rate (TR) for the clustering results obtained using kernel k-means with three different kernel functions (Gaussian, polynomial, and tangent) along with majority voting, and the proposed weighted majority voting based on average Silhouette.

DATA		Gaussian	Polynomial	Tangent	Majority Voting	Weighted Majority Voting
Bensaid	ASI	0.159	0.453	−0.127	0.132	0.167
	TR	0.528	0.641	0.413	0.592	0.611
DUNN	ASI	0.269	0.418	−0.003	0.293	0.293
	TR	0.453	0.521	0.417	0.474	0.474
IRIS	ASI	0.609	0.609	−0.068	0.482	0.589
	TR	0.856	0.850	0.381	0.833	0.873
SEED	ASI	0.594	0.601	−0.052	0.537	0.570
	TR	0.869	0.842	0.376	0.852	0.862

## Data Availability

The data that support the findings of this study are available from the corresponding author upon reasonable request.
